# Performance of Plug-In Augmented ChatGPT and Its Ability to Quantify Uncertainty: Simulation Study on the German Medical Board Examination

**DOI:** 10.2196/58375

**Published:** 2025-03-21

**Authors:** Julian Madrid, Philipp Diehl, Mischa Selig, Bernd Rolauffs, Felix Patricius Hans, Hans-Jörg Busch, Tobias Scheef, Leo Benning

**Affiliations:** 1 Department of Cardiology, Pneumology, Angiology, Acute Geriatrics and Intensive Care, Ortenau Klinikum, Klosterstrasse 18, Lahr, 77933, Germany, 49 7821932403; 2 Faculty of Medicine, University of Freiburg, Freiburg, Germany; 3 G.E.R.N. Research Center for Tissue Replacement, Regeneration and Neogenesis, Department of Orthopedics and Trauma Surgery, University of Freiburg, Freiburg, Germany; 4 University Emergency Center, Medical Center, University of Freiburg, Freiburg, Germany; 5 Department of Diagnostic and Interventional Radiology, Medical Center, University of Freiburg, Freiburg, Germany

**Keywords:** medical education, artificial intelligence, generative AI, large language model, LLM, ChatGPT, GPT-4, board licensing examination, professional education, examination, student, experimental, bootstrapping, confidence interval

## Abstract

**Background:**

The GPT-4 is a large language model (LLM) trained and fine-tuned on an extensive dataset. After the public release of its predecessor in November 2022, the use of LLMs has seen a significant spike in interest, and a multitude of potential use cases have been proposed. In parallel, however, important limitations have been outlined. Particularly, current LLMs encounter limitations, especially in symbolic representation and accessing contemporary data. The recent version of GPT-4, alongside newly released plugin features, has been introduced to mitigate some of these limitations.

**Objective:**

Before this background, this work aims to investigate the performance of GPT-3.5, GPT-4, GPT-4 with plugins, and GPT-4 with plugins using pretranslated English text on the German medical board examination. Recognizing the critical importance of quantifying uncertainty for LLM applications in medicine, we furthermore assess this ability and develop a new metric termed “confidence accuracy” to evaluate it.

**Methods:**

We used GPT-3.5, GPT-4, GPT-4 with plugins, and GPT-4 with plugins and translation to answer questions from the German medical board examination. Additionally, we conducted an analysis to assess how the models justify their answers, the accuracy of their responses, and the error structure of their answers. Bootstrapping and CIs were used to evaluate the statistical significance of our findings.

**Results:**

This study demonstrated that available GPT models, as LLM examples, exceeded the minimum competency threshold established by the German medical board for medical students to obtain board certification to practice medicine. Moreover, the models could assess the uncertainty in their responses, albeit exhibiting overconfidence. Additionally, this work unraveled certain justification and reasoning structures that emerge when GPT generates answers.

**Conclusions:**

The high performance of GPTs in answering medical questions positions it well for applications in academia and, potentially, clinical practice. Its capability to quantify uncertainty in answers suggests it could be a valuable artificial intelligence agent within the clinical decision-making loop. Nevertheless, significant challenges must be addressed before artificial intelligence agents can be robustly and safely implemented in the medical domain.

## Introduction

The GPT—recently updated to its fourth iteration (GPT-4)—is a generative and autoregressive large language model (LLM). It is pretrained on a vast corpus of internet text and fine-tuned on a labeled dataset using a transformer architecture [1-3]. GPT generates coherent and contextually appropriate text. It likely discovered a semantic grammar of language (ie, semantic regularities), enabling it to construct semantically and syntactically correct sentences [4,5]. However, GPT does not perform meaningful computations on symbolic representations [4-8]. The Wolfram language, a Turing-complete computational language, in contrast, allows such symbolic representation. GPT and the Wolfram language combined hence cover 2 different aspects of human cognition [4,9,10]. Combining these features, particularly when computation and symbolic representations are needed, represents a significant step toward general artificial intelligence (AI). This combination has already been successfully used to examine contradictions in Einstein Special Theory of Relativity equations [11].

In the light of these technological advances, LLMs show increasing promise in supporting medical training and practice. However, the models must acquire an in-depth and accurate representation of medical knowledge to be used in these sensitive domains. A medical board examination exemplifies these domains well, as it determines the qualification of medical students to obtain their license to practice medicine.

Our primary outcome is the model’s ability to achieve the minimum required score for passing the 2 written parts of the German medical licensing examination. This task poses a different challenge to an LLM than medical board examinations in the English language [12,13], as the performance of such models in other languages and in combination with more recent GPT versions and available plugins has not been explored. In the medical field, where mistakes can have severe consequences, assessing the amount of uncertainty is of paramount importance [14]. It is therefore crucial to gain insights into the depth and structure the LLMs have of the medical knowledge representation and where its limitations lie [15]. Hence, our secondary outcomes were the total correct answer rates, the presence of logical justification of the answer, the presence of information internal to the question, the presence of information external to the question, the confidence GPT displays in its answers, the difficulty of the question, information errors, logical errors, reasoning errors, and the correctness of a second try answer when the first answer was wrong. Insights into these 2 dimensions of outcomes can contribute to facilitating a meaningful use of novel LLM technologies in the medical domain.

## Methods

### Medical Board Examination Dataset

The German medical board examination consists of 3 steps. The first board examination, taken after 2 years of study, primarily covers basic natural sciences. It comprises 320 questions, which students answer over 2 consecutive days. The second board examination takes place after 6 years of study. It likewise consists of 320 medical questions, which students answer over 3 consecutive days. The third board examination, also after 6 years of study, is an oral examination and was, hence, excluded from this study. The German medical board examination takes place biannually, once in spring and once in fall. As a representative sample, we used the medical board examination from spring 2023. We excluded questions the medical board examination committee deemed inconsistent with the medical literature in the regular post examination review of the content. Additionally, we did not include questions displaying images, as GPT models could not analyze them at the time of our analysis. Furthermore, LLMs are not able to analyze images, GPT4vision which became broadly accessible in the second half of 2023 combines computer vision algorithms—which generate a text description of images—and LLMs to analyze this text. All questions and answers were exported from AMBOSS SE, a German medical education content creator and service provider.

### GPT Models and Prompt Engineering

We evaluated several GPT models with varying characteristics using OpenAI’s web interface. The models tested included GPT-3.5, GPT-4, GPT-4 integrated with the Wolfram, ScholarAI, and Web Request (WeGPT.ai) plugins, and GPT-4 integrated with the Wolfram, ScholarAI, Web Request plugins, and an additional feature for translating German inputs into English. We did not investigate earlier versions of GPT as they demonstrated lower performance in a similar study on the American medical board examination [12].

Creating a precise and adequate context is crucial for generating expected results [16,17]. Thus, we aimed to be as specific as possible, simulating the context of a medical student taking the medical board examination. The prompts hence included the request to answer each respective question with 5 possible answers, where only 1 answer was correct. We asked the models to justify their choices based on the provided patient case information, and to estimate their confidence in the answer’s accuracy as a percentage of maximal confidence (ie, 100%). If the selected answer was incorrect, the GPT models were asked to explain their mistake in a second attempt. For the GPT-4 model with plugin integration, we asked the model to use the available plugins (Wolfram, ScholarAI, and Web Request). For the GPT-4 model with plugin integration and English translation, we first asked the model to translate the input into the English language, and then to use the translated text to perform the abovementioned tasks. All used prompts are available in Multimedia Appendix 1.

### Model Testing and Outcome Parameters

For each GPT model, we used the appropriate prompt followed by the question and the possible answers. The investigators then analyzed the GPT’s answer to assess the defined primary and secondary outcomes, which were either binary or in proportions. In cases of uncertainty, the investigators (JM, TS, and LB) convened to resolve the issue.

First, the correctness of the answer was recorded (binary variable), followed by the presence of logical justification, the presence of information internal to the question, and the presence of information external to the question (binary variables).

Next, we recorded the model’s confidence in its answer (proportion), and the difficulty of the question, derived from the number of students who answered correctly on the AMBOSS platform (proportion).

To enhance our understanding of where GPT models falter, we sought to classify potential errors. As literature on error types is limited, we conducted a formal analysis to determine distinctive error types and established a formal definition. We propose a classification into 3 categories: information error, logical error, and reasoning error.

**Figure 1.**
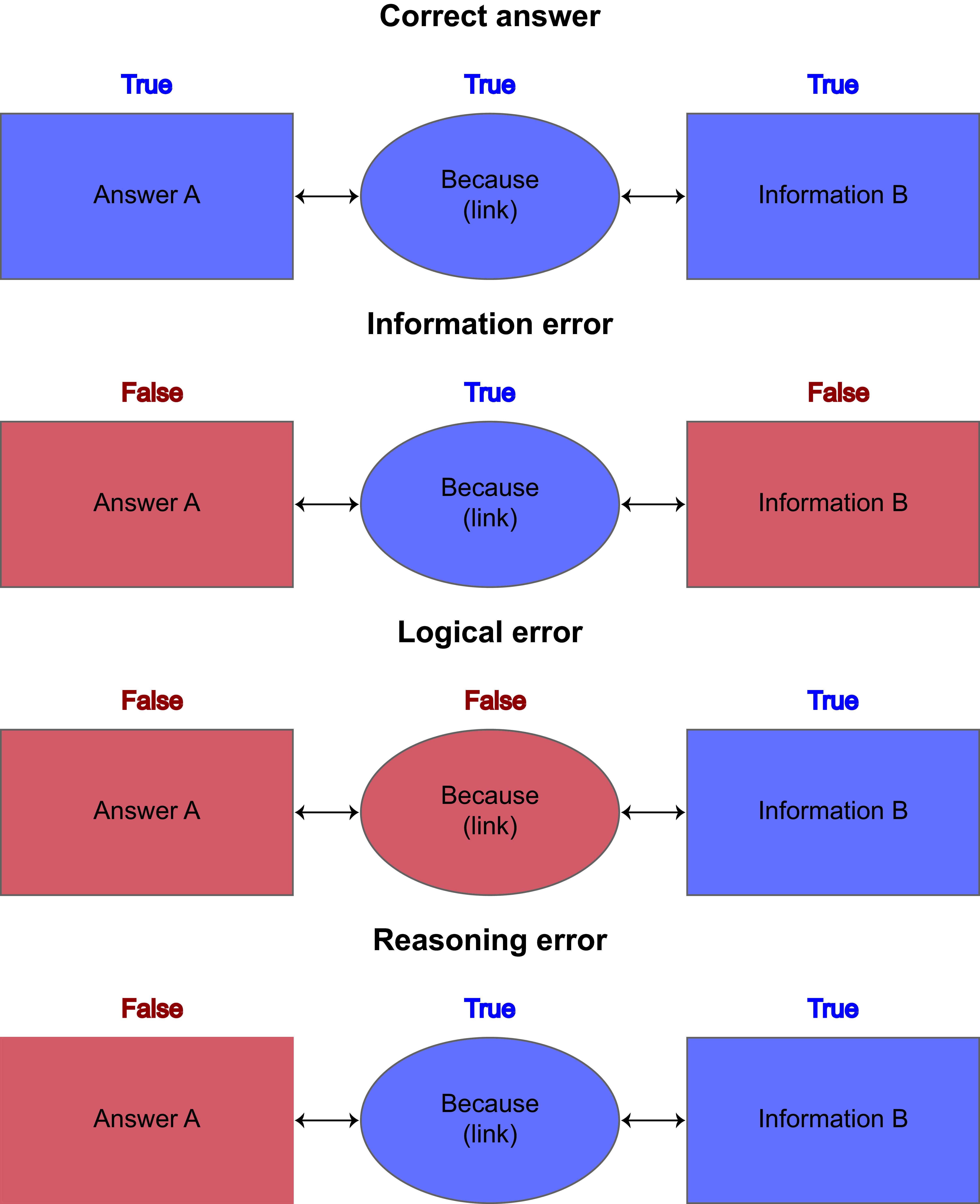
Formal definition of error types; we propose a classification into 3 categories: information error, logical error, and reasoning error. The GPT response can be formalized as “answer A” is given “link” because of “information B.” There are only three possibilities for errors: (1) “answer A” is incorrect because “information B” is incorrect—termed an information error; (2) “answer A” is incorrect while “information B” is correct, but the link between them is incorrect—termed a logical error; and (3) “answer A” is incorrect, “information B” is correct, and the link between “answer A” and “information B” is correct—termed a reasoning error.

The GPT response can be formalized as “answer A” is given “link” because of “information B.” There are only three possibilities for errors: (1) “answer A” is incorrect because “information B” is incorrect—termed an information error; (2) “answer A” is incorrect while “information B” is correct, but the link between them is incorrect—termed a logical error; (3) “answer A” is incorrect, “information B” is correct, and the link between “answer A” and “information B” is correct—termed a reasoning error (Figure 1). If the answer provided was incorrect, the investigator informed the GPT of its faulty answer, recorded whether it understood its mistake, and provided the correct answer in a second attempt. In the models with integrated plugin use, the active use of plugins was documented for Wolfram, ScholarAI, and Web Requests (binary variables).

### Data Analysis

Summary statistics were calculated for the outcome variables (Table 1 and MultimediaAppendices 2  3). Dichotomous variables were represented by frequency and proportions with 95% CIs, while continuous variables were expressed as mean values with 95% CIs. Uncertainty calculations displayed as 95% CIs were computed via bootstrapping [18].

**Table 1.** Characteristics of GPT model answers (N=541).

	GPT-3.5	GPT-4	GPT-4 + plugin	GPT-4 + plugin + translation
Correct answer (proportion±95% CI)	373 (0.69±0.65 to 0.73)	493 (0.91±0.89 to 0.93)	493 (0.91±0.89 to 0.94)	486 (0.9±0.87 to 0.92)
Logical justification (proportion±95% CI)	479 (0.89±0.86 to 0.91)	526 (0.97±0.96 to 0.98)	529 (0.98±0.96 to 0.99)	527 (0.97±0.96 to 0.99)
Question’s difficulty mean (±95% CI)	0.288 (0.272 to 0.303)	0.288 (0.272 to 0.303)	0.288 (0.272 to 0.303)	0.288 (0.272 to 0.303)
Error overall (proportion±95% CI)	168 (0.31±0.27 to 0.35)	48 (0.09±0.07 to 0.11)	48 (0.09±0.06 to 0.11)	55 (0.1±0.08 to 0.13)
Presence of internal information (proportion±95% CI)	521 (0.96±0.95 to 0.98)	537 (0.99±0.98 to 1)	537 (0.99±0.98 to 1)	537 (0.99±0.98 to 1)
Presence of external information (proportion±95% CI)	538 (0.99±0.99 to 1)	540 (1±0.99 to 1)	541 (1±1 to 1)	541 (1±1 to 1)
Information error (proportion±95% CI)	37 (0.22±0.16 to 0.29)	5 (0.1±0.02 to 0.19)	5 (0.1±0.02 to 0.2)	7 (0.13±0.05 to 0.22)
Logical error (proportion±95% CI)	61 (0.36±0.29 to 0.43)	18 (0.38±0.25 to 0.52)	12 (0.25±0.125 to 0.375)	19 (0.35±0.22 to 0.47)
Confidence mean (±95% CI)	0.912 (0.904 to 0.918)	0.938 (0.934 to 0.942)	0.919 (0.915 to 0.924)	0.919 (0.915 to 0.923)
Use of plugin Wolfram (proportion±95% CI)	N/A^a^	N/A	50 (0.09±0.07 to 0.12)	47 (0.09±0.06 to 0.11)
Reasoning error (proportion±95% CI)	72 (0.42±0.36 to 0.51)	26 (0.54±0.4 to 0.69)	30 (0.63±0.48 to 0.75)	29 (0.53±0.4 to 0.65)
Correct answer in second try (proportion±95% CI)	90 (0.54±0.46 to 0.61)	32 (0.67±0.52 to 0.79)	36 (0.75±0.63 to 0.88)	33 (0.6±0.47 to 0.73)
Use of plugin ScholarAI (proportion±95% CI)	N/A	N/A	107 (0.2±0.16 to 0.23)	47 (0.09±0.06 to 0.11)
Use of plugin web requests (proportion±95% CI)	N/A	N/A	2 (0.003±0 to 0.01)	25 (0.05±0.03 to 0.06)

a N/A: not applicable.

The primary outcome was determined by comparing the performance of the GPT-4 model, integrated with the plugins and the English translation, to the required passing score for the medical board examination, which is 60%. The difference of proportions was calculated with 95% CI using bootstrapping (Multimedia Appendix 4).

Subsequently, secondary outcomes were calculated: the final examination rate for each GPT model was compared to both chance and the required passing score for the medical board examination. The difference of proportions was calculated with 95% CI using bootstrapping (Multimedia Appendix 4).

The proportions of logical justification within the answer, information internal to the answer, and information external to the answer were compared between correct and incorrect responses. The difference of proportions was calculated with 95% CI using bootstrapping (Table 2 and Multimedia Appendix 5).

**Table 2.** Analysis of plugin-integrated GPT-4 model answers.

			All correct answers (n=493)	All incorrect answers (n=48)	Difference in proportions or Cohen *d* or Pearson *r* (±95% CI)	Confidence accuracy (±95% CI)
Comparison of GPT models justifications between correct and incorrect answers						
	GPT-4 + plugin (N=541)					
		Logical justification (proportion ±95% CI)	493 (1±1 to 1)	36 (0.75±0.63 to 0.88)	0.25 (0.13 to 0.38)^a^	—^b^
		Internal information (proportion ±95% CI)	489 (0.99±0.983 to 998)	48 (1±1 to 1)	0 (-0.01 to 0)^a^	—
Comparison of GPT models justifications between correct and incorrect answers						
		External information (proportion ±95% CI)	493 (1±1 to 1)	48 (1±1 to 1)	0 (0 to 0)^a^	—
Confidence of GPT models compared between correct and incorrect answers						
	GPT-4 + plugin (N=541)					
		Confidence mean (±95% CI)	0.923 (0.918 to 0.928)	0.886 (0.87 to 0.901)	-0.69 (-0.99 to -0.39)^c^	0.037 (0.021 to 0.053)
Comparison of question’s difficulty of GPT models between correct and incorrect answers						
	GPT-4 + plugin (N=541)					
		Question’s difficulty mean (±95% CI)	0.279 (0.263 to 0.295)	0.379 (0.327 to 0.438)	0.57 (0.27 to 0.86)^c^	—
Correlation of confidence and question’s difficulty for all answers						
	GPT-4 + plugin (N=541)				-0.0874 (-0.176 to 0.004)^d^	
		Confidence mean (±95% CI)	0.920 (0.916 to 0.924)	—		—
		Question’s difficulty mean (±95% CI)	—	0.288 (0.273 to 0.304)		—
Comparison of correct answers between GPT models (N=541)						
	GPT-4 + plugin vs GPT-3.5					
		Correct answer rate (proportion ±95% CI)	373 (0.69±0.65 to 0.73)	493 (0.91±0.89 to 0.94)	0.22 (0.18 to 0.27)^a^	—
	GPT-4 + plugin vs GPT-4					
		Correct answer rate (proportion ±95% CI)	493 (0.91±0.89 to 0.94)	493 (0.91±0.89 to 0.94)	0 (-0.03 to 0.03)^a^	—
	GPT-4 + plugin vs GPT-4 + plugin + translation					
		Correct answer rate (proportion ±95% CI)	493 (0.91±0.89 to 0.94)	486 (0.9±0.87 to 0.92)	-0.01 (-0.05 to 0.02)^a^	—

a Difference in proportions.

b Not available.

c Cohen *d*.

d Pearson *r*.

The model’s confidence in its answers was compared between correct and incorrect responses. Additionally, the relationship between the model’s confidence in its answers and the difficulty of the question was assessed. Cohen *d* values and 95% CI were computed using a linear regression model and bootstrapping (Table 2 and MultimediaAppendices 6  7).

To evaluate the accuracy of the model’s confidence in its answers, we developed a parameter termed confidence accuracy (CA). It is defined as follows:

CA = (confidence of correct answers in percentage – confidence of incorrect answers in percentage)/100

Consequently, this parameter can take values from −1 to 1, where 1 accurately reflects the model’s uncertainty, 0 indicates no ability to quantify uncertainty, and −1 suggests incorrect quantification.

The difficulty of the question was assessed using real correct response proportions from students available on the AMBOSS platform. The difficulty was assessed as follows:

Difficulty=1 – correct answer proportion

Then, the difficulty of the question was compared between correct and incorrect answers, with Cohen *d* calculated using a linear regression model (Table 2 and Multimedia Appendix 7).

Furthermore, we compared the proportion of correct answers between models (Table 2 and Multimedia Appendix 8).

We compared the proportion of correct answers in the GPT-4 models with the proportion of correct answers in the answers where a plugin has been used. We compared the proportion of plugin usage in GPT models with German and English input. We compared the confidence of the model when using plugins to the confidence of the model overall. We compared the proportion of correct answers when averaging the 4 different models to each model in particular (Multimedia Appendix 9).

In instances where questions were accompanied by images, GPT models sometimes responded by describing the image, although the models could not access the respective images. This phenomenon is known as a type of hallucination [19]. Therefore, we compared the proportion of hallucinations present in each model when answering questions, including image questions. We calculated the proportion of correct answers for each model when keeping the questions with pictures (Multimedia Appendix 9).

We compared the different error proportions between different models. We compared the proportion of logical errors when using the Wolfram plugin to the proportion of errors when using the entire model. We compared correct second-try answers between different models (Multimedia Appendix 9).

The 95% CIs were calculated using bootstrapping. Where necessary, parametric assumptions were tested using quantile-quantile plots for normality and Levene tests for the homogeneity of variances. The independence of question answers was assumed. All statistical analyses were performed in RStudio (version 2023.06.0+421). The significance level for all tests was set a priori at 95% CI.

## Results

All tests were performed on the 541 questions of the German medical board examination from spring 2023. Sub analyses were performed on other subgroups, the respective sample sizes are indicated in the appropriate tables. All results for GPT-3.5, GPT-4, GPT-4 + plugin (GPT4P), and GPT-4 + plugin + translation (GPT4PT) are listed in full detail in the tables and the supplementary materials. To ensure legibility, only relevant results are addressed in the results section.

Descriptive statistics with CIs for the first board examination, second board examination, and the overall examination are displayed in Table 1 and MultimediaAppendices 2  3.

All models performed significantly better than chance. Furthermore, all GPT models were significantly better than the required proportion to pass the final medical board examination.

All GPT models had a significantly higher proportion of providing a logical justification for correct answers compared to incorrect answers (Table 2 and Multimedia Appendix 5). Yet, there was no statistical significance for the proportion of used internal information for correct and incorrect answers (Table 2 and Multimedia Appendix 5). Similarly, there was no statistical significance for the proportion of used external information for correct and incorrect answers (Table 2 and Multimedia Appendix 5).

Although generally high for both incorrect and correct answers, models had a confidence mean which was significantly higher for correct answers than incorrect answers (Table 2 and Multimedia Appendix 6). This is reflected in CA values significantly different from zero: GPT-3.5 (0.028, 95% CI 0.011 to 0.048), GPT-4 (0.041, 95% CI 0.023 to 0.062), GPT4P (0.037, 95% CI 0.021 to 0.053), and GPT4PT (0.043, 95% CI 0.028 to 0.059).

From all models, only GPT4P made significantly more reasoning errors than logical errors (0.37, 95% CI 0.125 to 0.60). All models made significantly more reasoning errors than information errors: GPT-3.5 (0.21, 95% CI 0.11 to 0.30), GPT-4 (0.44, 95% CI 0.27 to 0.60), GPT4P (0.52, 95% CI 0.31 to 0.71), and GPT4PT (0.40, 95% CI 0.20 to 0.58). All models but GPT4P made significantly more logical errors than information errors: GPT-3.5 (0.14, 95% CI 0.029 to 0.26), GPT-4 (0.27, 95% CI 0.10 to 0.44), and GPT4PT (0.22, 95% CI 0.05 to 0.38). GPT-4 (0.12, 95% CI 0.05 to 0.22) and GPT4P (0.12, 95% CI 0.02 to 0.22) made significantly less information errors than GPT3.5.

The GPT4-based models all performed better than the GPT 3.5 model in providing correct answers as reflected in the difference of correct answer proportions (Table 2 and Multimedia Appendix 8). However, no GPT4-based model was better than another GPT4-based model, as reflected in the difference of correct answer proportions (Table 2 and Multimedia Appendix 8).

## Discussion

### Primary Outcome

All GPT models assessed performed above the minimum required score of 60%. The GPT-4 models performed particularly well, outperforming most students in the given examinations. Specifically, for the first board examination, all GPT-4 models performed better than 98.6% of students. For the second board examination, they surpassed 95.8% of students, as detailed in the records of the examining body [20].

In general, there was a significant gap between GPT-3.5 and the GPT-4 models. The more recent models, with substantially more parameters and the capacity to remember longer prompts, appear to increase the accuracy of responses. However, we observed no additional benefit when GPT-4 models were paired with plugins.

The use of plugins did not yield a higher proportion of correct answers than the standard model. It is possible that GPT-4 already achieves a very high rate of accuracy, resulting in a ceiling effect. Hence, the addition of plugins may not offer a significant advantage for the questions prompted.

During our study, we noted that the Wolfram plugin was frequently used for more complex calculations. Yet, in the context of clinically applicable questions, complex mathematical procedures are typically not required and the use of symbolic language is usually not required. Thus, using the Wolfram Alpha plugin is likely more beneficial for questions that involve extensive computations or advanced mathematical problems requiring symbolic representations. The ScholarAI plugin was activated for complex informational queries, but the resulting papers were not consistently useful. Surprisingly, the Internet Access plugin (WeGPT.ai) was the least used. This may be because answering medical questions typically demands expert-level knowledge, and general internet searches do not provide sufficiently specific information. Moreover, since the model has been trained on a vast amount of internet data, it likely already encompasses the knowledge available from the world wide web within its parameters.

We speculated that posing questions in German might hinder the model’s access to the broader body of knowledge available in English. However, this was not the case; the GPT model equipped with translation capabilities did not outperform the GPT-4 models without translation features. The GPT model likely abstracts high-level concepts and is not impeded by the language of the queries. This aligns with the LLMs’ transformer architecture, which accesses higher-level concepts prior to translating text into another language [21].

Interestingly, the GPT-4 model with translation invoked plugins less frequently than the model without translation. We hypothesize that plugin calls occur at a lower level in the neural network, making them less necessary in English due to the larger available language corpus. In German, the model might need to delve deeper into the latent representation of concepts not tied to a specific language. However, this remains speculative and warrants further research.

### Secondary Outcomes

While all models provided a very high proportion of logical justification for correct answers, it was significantly less extensive for incorrect answers. However, upon further analysis, we did not detect a significant difference in the proportion of internal information from the question in the answer or in the use of external information not contained in the question between correct and incorrect answers. One study already assessed the presence of logical justification in answers to United States Medical Licensing Examination questions, where all answers exhibited logical justification regardless of their accuracy [12]. Hence, this metric could not be used as a discriminator for correctness.

We were unable to demonstrate a significant correlation between the model’s confidence in an answer and the difficulty level of the question for humans. This suggests that the model’s interpretation of question difficulty differs from that of humans. However, as with humans, the model showed improved performance on easier questions compared to more challenging ones. Thus, it appears that the representation of question difficulty is distinct between LLMs and humans.

### Conceptual Implications

#### Use for Medical Education

This performance suggests that LLMs such as GPT could assume a greater role in medical education, as their integration could significantly change the conventional approach to medical education, which has traditionally emphasized the acquisition and maintenance of medical knowledge. The emergence of AI agents with superior information retention abilities, however, prompts a reevaluation of our educational focus. In this light, teaching methodologies could shift toward navigating and structuring available information with respective AI agents. The approach could hence shift from retaining information to learning how to efficiently access information and deeply understand these systems, along with their benefits and drawbacks.

#### Use in Clinical Practice

The utility of LLMs is not limited to educational settings but also extends to clinical practice. Although LLMs may not be as effective in highly specialized tasks where dedicated machine learning algorithms excel—for instance, XGBoost in identifying pulmonary embolisms [22-24] — LLMs are highly proficient in text processing and information integration from diverse algorithms [25]. This positions them as intelligent medical assistants, capable of transforming complex data into narratives that are comprehensible in a human context. Currently, clinicians have a limited understanding of AI agents and their functions. Clinicians must, therefore, gain a thorough understanding of how various AI agents function, including their strengths and weaknesses.

With insufficient knowledge on the principles of LLM-based assistants, clinicians are at risk of blindly following such assistant’s guidance without fully understanding its operations [26,27]. Due to the inherent complexity of LLMs, which often function as a black box, we can only partially monitor their operations at varying levels of complexity and behavior [26]. Given the marginal uncertainty intrinsic to such complex models, the AI agent should not supplant clinicians in decision-making, but rather provide additional informed perspectives.

To serve as a useful assistant, however, the assessment of uncertainty for any output provided by such is crucial. The key attribute enabling this evaluation is the ability to quantify uncertainty, a trait humans are presumed to possess [14]. For LLM-based assistants to provide a comparable estimate, a standardized measure is needed to gauge the confidence in an AI agent’s output. For binary outcomes such as healthy or diseased, metrics such as specificity, sensitivity, and area under the curve are effective. For more complex queries with multiple potential answers—as managed by LLMs—traditional measures such as sensitivity and specificity are inadequate. We therefore developed a new metric called “confidence accuracy” (CA) which correlates the confidence assigned to an answer with its empirical accuracy. This allows for the quantification of uncertainty, crucial for clinical decision-making. Although our work showed that all GPT models have the ability to quantify uncertainty, the expression in percentage does not seem to reflect the confidence for any specific decision (ie, the models were overall largely overconfident). Although statistically different from zero, CA values were consistently close to zero. New LLM methodologies aim to enhance this by incorporating uncertainty estimation [28]. Future AI agents should be fine-tuned using the CA metric in order to improve uncertainty quantification, a critical objective for implementing AI as a supportive tool for physicians in clinical environments.

#### Identified Errors

We observed that GPT models commit different types of errors, particularly reasoning errors. Reasoning errors typically occur in situations where multiple options are correct, but one is more critical than the other. GPT models over proportionately make reasoning errors likely because this skill is acquired through human experience and is challenging to learn from text-based web sources. The second most common error type in GPT models was logical errors. Since LLMs use a statistical approach to reconstruct human-written text, we anticipated difficulties with logic and mathematics, which require formal symbolic representation [4-8]. We hypothesized that the Wolfram plugin, using the Wolfram language, would mitigate these challenges. Yet, using the Wolfram plugin did not reduce the number of logical errors. Finally, fewer information errors were observed compared to other error types across all GPT models. This likely reflects the strength of these LLMs, which have assimilated a vast corpus of knowledge. In addition to the 3 error types derived from the informational and logical structure of GPT’s answers, there are 2 sources of bias that arise prior to answer generation. First, due to the stochastic nature of token generation, there is likely a stochastic bias inherent in all GPT responses. Second, due to in-context generation conditioned by the prompting strategy, a systematic bias probably occurs as well. We attempted to mitigate the stochastic bias by averaging the results from all models and selecting the most common outcome. However, the performance of such averaged models did not surpass that of the GPT-4 models.

To assess whether the GPT models could recognize and correct their own mistakes, we prompted them to attempt another answer after providing incorrect responses. In most instances, the model would acknowledge the mistake and provide the correct answer along with a new explanation. This phenomenon could likely be attributed to the differing mechanics of forward and backward reasoning in LLMs. With forward reasoning, the LLM calculates the probability of the next token without a specific reasoning goal [29]. In contrast, backward reasoning enables the LLM to better contextualize the information. It is crucial to note, however, that we did not request the model to immediately reassess the answer; instead, we informed it of the answer’s incorrectness before asking for a reevaluation [29]. Future studies could further investigate the model’s ability to self-correct without prior notification of its errors.

In instances where questions were accompanied by images (ie, the model did not have access to the images), GPT models, particularly GPT-3.5, often responded by describing the image that the model had not actually seen. This unexpected information error, known as a hallucination [19], persisted in the GPT-4 models, albeit at a significantly reduced frequency compared to GPT-3.5. Nevertheless, the propensity for overconfidence in entirely fabricated information remains a challenge for the latest generation of LLMs and is a phenomenon not fully understood [30].

### Limitations

#### Technological Limitations of LLMs

Although the results were impressive with GPT outperforming most students in the German medical board examination, it is crucial to remember that these models still possess significant limitations. At the time of our data collection, GPT-4 was incapable of interpreting medical images, such as chest x-rays or histological samples. This is a considerable drawback, given that medical information is inherently multimodal, and the ability to integrate multimodal data will be essential for the adoption of such models in academic and clinical settings. It is anticipated that future GPT iterations and other LLMs will be fully multimodal, which necessitates additional research to evaluate their performance across a more diverse array of questions.

A second concern relates to the stochastic nature of token generation, meaning that answers may vary slightly when questions are posed multiple times [31].

A third concern pertains to the prompt sensitivity of LLMs. This trait can be advantageous as it allows the incorporation of context into the generation of meaningful output and may contribute to the models’ Bayesian characteristics [32]. However, prompt sensitivity also increases the risk of systematic errors with repetitive use of the same prompt. Prompt engineering is a discipline that emerged in trying to minimize systematic errors [33,34].

Within the extensive volume of data available online, there are significant risks of bias. Given that LLMs are trained on vast datasets, there is an inherent risk of adopting biases from the underlying data structures. However, fine-tuning through supervised learning on labeled data can help mitigate these risks [35,36].

#### Limitations of the Use of LLMs in a Medical Context

Despite the seemingly immediate promise of using LLMs in both educational and clinical contexts, the current ethical and regulatory environment needs to be considered to advance the use of these novel technologies safely.

As the representation of medical information of an LLM must not be confused with medical knowledge from a medical professional, it remains crucial to enable students and medical professionals alike to identify LLM-generated outputs as such in order to interpret them very carefully. Different to, for example, a senior medical colleague providing guidance for a clinical decision, an LLM-generated output is neither based on clinical knowledge, nor experience. The risk of such confusion has been described as anthropomorphic projection and efforts for advancing these novel technologies in the medical field need to simultaneously foster the awareness of such phenomena. This differentiation resonates with the provisions of the European Union (EU) on a risk-based assessment approach [37] and, more recently, with the Bletchley Declaration [38]. The latter emphasizes the risks at the “frontier” of AI, at which we operate with the presented project.

While the concerns discussed in the context of medical education—and, more widely, training—are mainly within the realm of AI ethics, more specific limitations apply to the clinical use of these technologies. At the time of our analysis, no commercially available LLM in the EU—including the GPT versions assessed in this work—have an assigned intended medical use, a basic regulatory prerequisite for their use in a clinical context. Without such intended medical use, the Medical Device Regulation (MDR), the regulatory framework for medical devices in the EU, is not applicable. Hence, such a device would not be a medical device in the regulatory sense and could, therefore, not be used in a clinical context without irresponsible safety and liability risks. While it is not the user (eg, researchers or clinicians), but the manufacturer (eg, OpenAI for the ChatGPT models) who assigns an intended medical use—which itself comes with further regulatory requirements—the clinical use of the currently available and mostly all-purpose LLMs remains challenging.

Yet, even developing an LLM with an intended medical use and fulfilling all adjacent regulatory requirements would—as of now—not necessarily resolve the challenge centering around the clinical use of such program, as a key requisite for software as a medical device outlined in the MDR (“devices that incorporate electronic programmable systems, including software, or software that are devices in themselves, shall be designed to ensure repeatability, reliability and performance in line with their intended use.” MDR Annex I, Rule 17.1 [39]) is currently considered to be violated, although this question remains subject to debate.

However, the rapid development of technological advances and the concurrent establishment of respective regulations should not be perceived as a “race to get to grips with AI” [40], but should be viewed as a co-evolution to eventually yield the best population-wide benefit from these technological advances. In this light, the emphasis of a “pro-innovation and proportionate governance,” as proposed in the Bletchley Declaration, is equally as crucial as the implementation of regulatory frameworks.

#### Limitations of This Study

Our study has several limitations. We used a specific German medical board examination as a sample to represent the general distribution of medical questions. While it is acknowledged that questions evolve over time and may introduce bias, the objective of the medical board examination is to maintain a consistent level of difficulty, reflecting the minimum required knowledge to attain board approval for medical practice. The distribution of student grades has remained relatively stable over time, leading us to believe that this potential bias is minimal. In the model with translation, we used GPT to translate the questions before applying them to the model. Although we did not observe any, it is possible that translation errors occurred, potentially acting as a confounder in this study. In the context of the medical board examination, multiple-choice questions are posed to elicit clear answers that can be quantitatively assessed. By contrast, in a clinical setting, questions tend to be open-ended, which introduces a different dynamic. Nevertheless, we asked the model to justify its answers to glean insight into its computational process, thus rendering the questions more comparable to open-ended inquiries.

### Conclusion

The performance of GPT models in the German medical board examination have surpassed both the passing threshold and the performance of most students. While GPT appears to possess a latent representation of uncertainty, it currently exhibits a significant degree of overconfidence. The introduced metric of CA could facilitate the appropriate measurement and fine-tuning of models to improve this aspect. However, there are numerous limitations that clinicians should be aware of. Challenges such as hallucinations, the stochastic nature of token generation, and prompt sensitivity are highlighted, indicating areas for further research and development. Further, we see the remaining open questions regarding the ethical and regulatory use of LLMs in the educational and clinical context, which need to be addressed on a policy level.

## Supplementary material

10.2196/58375 Multimedia Appendix 1 Prompting strategies for different GPT models.

10.2196/58375 Multimedia Appendix 2 Question’s difficulty and error structure of GPT model answers.

10.2196/58375 Multimedia Appendix 3 Correct answers of GPT models compared with required and random scores.

10.2196/58375 Multimedia Appendix 4 Comparison of correct answers between GPT models.

10.2196/58375 Multimedia Appendix 5 Supplementary analysis of GPT models answers (statistically significant results are highlighted in blue and statistically nonsignificant results are highlighted in brown).

10.2196/58375 Multimedia Appendix 6 Confidence of GPT models compared between correct and incorrect answers.

10.2196/58375 Multimedia Appendix 7 Relationship between question’s difficulty, performance, and confidence of GPT model answers.

10.2196/58375 Multimedia Appendix 8 Comparison of GPT models justifications between correct and incorrect answers.

10.2196/58375 Multimedia Appendix 9 Performance, information content, confidence, and plugin usage of GPT model answers.

## Abbreviations

AI: artificial intelligence
CA: confidence accuracy
EU: European Union
GPT4P: GPT-4 + plugin
GPT4PT: GPT-4 + plugin + translation
LLM: large language model
MDR: Medical Device Regulation
