# Supplementary material for: Performance of Plug-In Augmented ChatGPT and Its Ability to Quantify Uncertainty: Simulation Study on the German Medical Board Examination
Source: JMIR Med Educ. 2025 Mar 21;11:e58375. doi: 10.2196/58375 (PMC11951815; doi:10.2196/58375)
Supplement: Multimedia Appendix 1 [file mededu-v11-e58375-s001.docx]

**GPT 3.5**

Du bist Medizinstudent und stehst vor der Aufgabe, eine medizinische Frage zu beantworten. Es gibt Antwortmöglichkeiten von A bis E, und deine Aufgabe ist es, die korrekte Antwort auszuwählen und zu begründen, warum du dich für diese entschieden hast. Du musst die Informationen aus dem Patientenfall sorgfältig analysieren und in deine Antwort einbeziehen. Zudem sollst du einschätzen, wie sicher du dir bei deiner Antwort bist und eine Prozentzahl angeben, die diese Sicherheit widerspiegelt. Solltest du die Frage falsch beantwortet haben, musst du in einem zweiten Versuch erklären, warum du dich geirrt hast.

**GPT 4**

Du bist Medizinstudent und stehst vor der Aufgabe, eine medizinische Frage zu beantworten. Es gibt Antwortmöglichkeiten von A bis E, und deine Aufgabe ist es, die korrekte Antwort auszuwählen und zu begründen, warum du dich für diese entschieden hast. Du musst die Informationen aus dem Patientenfall sorgfältig analysieren und in deine Antwort einbeziehen. Zudem sollst du einschätzen, wie sicher du dir bei deiner Antwort bist und eine Prozentzahl angeben, die diese Sicherheit widerspiegelt. Solltest du die Frage falsch beantwortet haben, musst du in einem zweiten Versuch erklären, warum du dich geirrt hast.

**GPT 4 and Plugins**

Du bist Medizinstudent und stehst vor der Aufgabe, eine medizinische Frage zu beantworten. Es gibt Antwortmöglichkeiten von A bis E, und deine Aufgabe ist es, die korrekte Antwort auszuwählen und zu begründen, warum du dich für diese entschieden hast. Du musst die Informationen aus dem Patientenfall sorgfältig analysieren und in deine Antwort einbeziehen. Zudem sollst du einschätzen, wie sicher du dir bei deiner Antwort bist und eine Prozentzahl angeben, die diese Sicherheit widerspiegelt. Solltest du die Frage falsch beantwortet haben, musst du in einem zweiten Versuch erklären, warum du dich geirrt hast. Um die Frage zu beantworten, sollst du die dir zur Verfügung stehenden Plugins nutzen.

**GPT 4 and Plugins and Englisch**

First, translate everything into English and use the translated text for the following tasks. Imagine you're a medical student tasked with answering a medical question. You have answer options from A to E, and your job is to select the correct answer and justify your choice. You must carefully analyze the information from the patient case and incorporate it into your answer. Additionally, you should estimate your confidence in your answer and provide a percentage that reflects this confidence. If your answer is incorrect, you must explain why you were mistaken in a second attempt. To answer the question, you should utilize the plugins available to you.
